# Hereditary angioedema in Spain: medical care and patient journey

**DOI:** 10.1186/s13023-024-03182-1

**Published:** 2024-05-21

**Authors:** Teresa Caballero, Carmen Alonso, María Luisa Baeza, Krasimira Baynova, José Cabeza, Isabel Cortés, Danilo Escobar Oblitas, Mar Guilarte, Alejandro Joral, Jesús Jurado Palomo, María Ángeles Lara Jiménez, Ana Martínez Virto, Laura Medrano, Emilio Monte Boquet, Montserrat Navarro, Diego Pérez, María José Plá Martí, Sara L. Smith Foltz, Coral Suero, Carolina Zamora

**Affiliations:** 1 Allergy department, Hospital Universitario La Paz, Madrid, Spain; 2 Hospital La Paz Institute for Health Research (IdiPAZ), Madrid, Spain; 3 Biomedical Research Network on Rare Diseases (CIBERER U754), Madrid, Spain; 4 Nursing department, Complejo Hospitalario Universitario de Vigo, Vigo, Pontevedra, Spain; 5 Allergy department, Complejo Hospitalario Universitario de Vigo, Vigo, Pontevedra, Spain; 6 Biomedical Research Network on Rare Diseases U761 (CIBERER), Madrid, Spain; 7 Allergy department, Hospital Universitario Virgen del Rocío, Madrid, Spain; 8 Hospital Pharmacy, Hospital Universitario Clínico San Cecilio, Granada, Spain; 9 Market Access, CSL Behring, Barcelona, Spain; 10 Immunology department, Hospital Universitario Son Espases, Palma de Mallorca, Illes Balears, Spain; 11 Health Research Institute of the Balearic Islands (IdISBa), Palma de Mallorca, Illes Balears, Spain; 12 Allergy department, Hospital Universitario Vall d’Hebron, Barcelona, Spain; 13 Allergy department, Hospital Universitario Donostia, Gipuzkoa, Spain; 14 Allergy department, Hospital General Universitario Nuestra Señora del Prado, Talavera de la Reina, Toledo Spain; 15 Allergy department, Hospital Universitario Clínico San Cecilio, Granada, Spain; 16 Emergency department, Hospital Universitario La Paz, Madrid, Spain; 17 Asociación Española De Angioedema Familiar, Torrelodones, Madrid Spain; 18 Hospital Pharmacy, Hospital Universitario y Politécnico La Fe, Valencia, Spain; 19 Hospital Pharmacy, Hospital Universitario Santa María, Lleida, 25198 Spain; 20 Nursing department, Hospital Universitario Virgen del Rocío, Sevilla, Spain; 21 Nursing department, Hospital Universitario y Politécnico La Fe, Valencia, Spain; 22 Emergency department, Hospital Regional Universitario de Málaga, Málaga, 29010 Spain

**Keywords:** Hereditary angioedema, Patient journey, Recommendations, Acute attacks, Short-term prophylaxis, Long-term prophylaxis, Quality of life

## Abstract

**Background:**

Hereditary angioedema due to C1 inhibitor deficiency (HAE-C1INH) is a genetic rare disease characterized by recurrent, transient and unpredictable episodes of cold, non-pruriginous oedema without associated urticaria. The characteristics of the disease have a considerable impact on the quality of life of patients. The aim of this study was to increase understanding of the patient journey of HAE in Spain.

**Methods:**

A multidisciplinary committee of 16 HAE experts (allergy, immunology, emergency department, hospital pharmacy and nursing) and 3 representatives of the Spanish Hereditary Angioedema Patient Association (AEDAF) who were patients or caregivers participated in the study. A review of the publications on HAE treatment was performed. Semi-structured interviews were performed to HAE experts, patients, or caregivers. Three meetings with the experts, patients and caregivers were held to share, discuss, and validate data obtained from literature and interviews and to build the model.

**Results:**

Throughout the project, the patient journey has been drawn up, dividing it into the stages of pre-diagnosis, diagnosis and treatment/follow-up. Some areas for improvement have been identified. Firstly, there is a need to enhance awareness and training on HAE among healthcare professionals, with a particular emphasis on primary care and emergency department personnel. Secondly, efforts should be made to minimize patient referral times to allergy/immunology specialists, ensuring timely access to appropriate care. Thirdly, it is crucial to encourage the study of the relatives of diagnosed patients to early identify potential cases. Fourthly, equitable access to self-administered treatments should be ensured, facilitated by systems that enable medication delivery at home and proper education and training for patients. Equitable access to long-term prophylactic treatment should also be prioritized for all patients in need. To standardize HAE management, the development of consensus guidelines that reduce variability in clinical practice is essential. Lastly, promoting research studies to enhance knowledge of the disease and align its treatment with new developments in the healthcare field should be encouraged.

**Conclusions:**

The knowledge of the patient journey in HAE allowed us to identify improvement areas with the final aim to optimize the disease management.

## Introduction

Hereditary angioedema due to C1 inhibitor deficiency (HAE-C1INH) is an autosomal dominant genetic disease caused by mutations in the *SERPING1* gene that encodes for C1 esterase inhibitor (C1INH) [1]. Its main form is type 1, characterized by a quantitative deficiency of C1INH [1].

It is a rare disease, of which its exact prevalence is unknown. In a systematic review of epidemiological studies published in 2018, its prevalence was estimated between 1.1 and 1.6 cases per 100,000 population [2]. One study analysed the disease prevalence in Spain with data collected between 2003 and 2004, calculating a minimum prevalence for HAE-C1INH of 1.09 per 100,000 population [3].

The clinical expression of HAE-C1INH is characterized by recurrent, transient and unpredictable episodes of cold, non-pruriginous oedema without associated urticaria [1, 4–6]. These acute attacks can appear in different locations (peripheral, abdominal, cervicofacial and upper respiratory tract oedemas) and resolve spontaneously within 2–5 days [1, 5, 6]. The frequency and severity of episodes vary greatly between patients, even when they are from the same family and share the same genetic mutation, and also in the same individual during the different life stages [1]. This wide heterogeneity makes it difficult to diagnose and manage [7, 8].

The diagnosis of hereditary angioedema (HAE) is based on clinical suspicion and the existence of a family history, and requires confirmation through the complement study (C4, C1q, C1 inhibitor, and functional C1 inhibitor) and the *SERPING1* gene study [8, 9]. Treatment of HAE-C1INH has evolved in recent years, and currently focuses on three therapeutic strategies that aim to prevent the onset of oedema, its progression, and morbidity and mortality: on-demand treatment of the acute attack, short-term prophylaxis (STP) and long-term prophylaxis (LTP) [5, 8, 10–12].

The characteristics of the disease (unpredictability of attacks and their severity, risk of asphyxia, need for urgent care, the hereditary nature of the disease and concerns about transmission to offspring, difficulty in accessing specific treatments and the adverse effects of a number of them) produce a high impact on the quality of life (QoL) [13]. Furthermore, its low prevalence and heterogeneity and the lack of awareness of the disease among healthcare professionals are associated with underdiagnosis, misdiagnosis and delayed diagnosis [14].

In this context, this study was developed with the aim of understanding the typical journey of a patient with HAE-C1INH in Spain, and the different profiles and clinical situations that may be encountered, and experienced in the different stages of the disease, in order to increase awareness about patients with this disease and improve its treatment.

## Methodology

An independent external consulting team from Ascendo Consulting Sanidad & Farma was in charge of coordinating and energizing all phases of the study. A multidisciplinary committee of experts composed of 16 professionals from different specialties with extensive experience in the treatment of HAE in Spain: allergy, immunology, emergency departments (ED), hospital pharmacy and nursing, as well as three representatives of the Spanish Patient Association of Hereditary Angioedema (AEDAF), who are themselves patients or caregivers, participated in the study. The panel represented different geographical areas of Spain with different health care systems.

The methodology employed consisted of: (1) a review of the literature on the diagnosis and treatment of HAE-C1INH through PubMed; (2) individual interviews with each of the members of the multidisciplinary committee using different semi-structured questionnaires for each profile, in order to supplement any information not available in literature sources and include their clinical experience; and (3) meetings with the entire multidisciplinary committee, including representatives of patients and caregivers, aimed at sharing, discussing and validating the conclusions obtained from the literature review and individual interviews. All the results expressed in the present study were approved with a minimum of 80% consensus among the panel of experts.

## Results

The expert surveys showed a high variability in the HAE-C1INH patient journey. Several teleconferences were held to discuss the results of the surveys and the literature review and build the HAE-C1INH patient journey. In those cases where there was a high variability or there were no published data, the experts were specifically asked to reach a consensus.

The HAE-C1INH patient journey was structured in three phases: pre-diagnostic phase; diagnostic phase; and treatment/follow-up phase after diagnosis.

The findings for each of these phases are shown below.

### Pre-diagnostic phase

HAE is an underdiagnosed disease in which the symptoms are often similar to other more common diseases and, as such, are not recognized as typical of the disorder [15]. Accordingly, diagnosis is usually late, although delays have decreased in recent years due to growing knowledge of the disease among healthcare professionals [15].

The diagnosis delay in Spain in different studies can be seen in Table 1.

**Table 1:** Mean diagnostic delay in HAE-C1INH in Spain

	Mean (SD)
Roche, et al. [3]	13.1 (± 15.2)
Caballero, et al. [16]	12.0 (± 15.0)
Gómez-Traseira, et al. [17]	8.5 (± 11.1)

According to Lunn et al. HAE-C1INH, patients attended an average of 4.4 different departments or clinics before receiving a correct diagnosis in a patient survey in the USA [18]. This was confirmed later in Japan by Iwamoto et al. that found that only 12% of patients were diagnosed with HAE at the first medical institution that they visited, and they attended an average of 4.6 ± 4.6 different areas of specialty before HAE diagnosis [19].

**Fig. 1:**
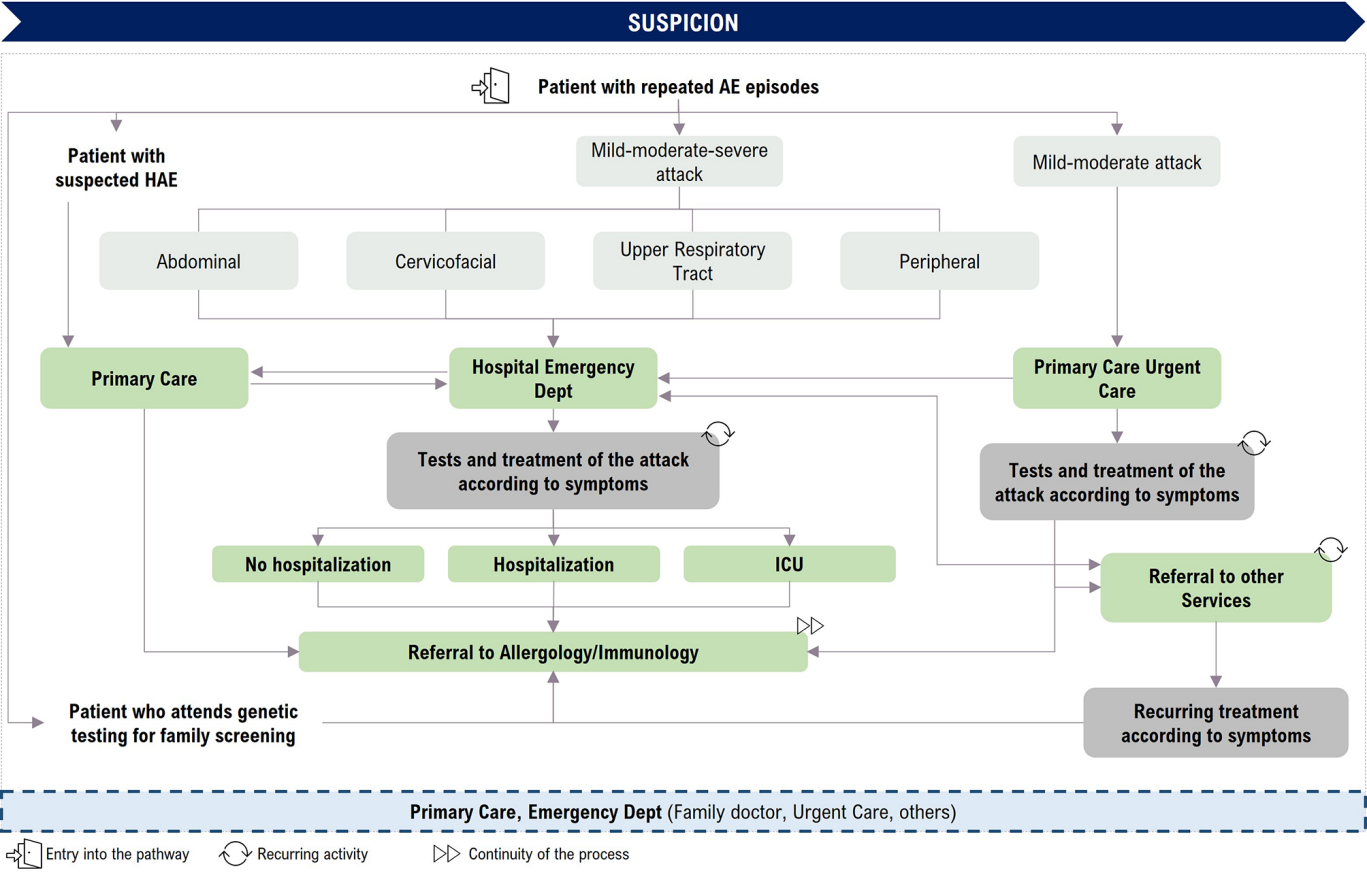
Patient journey: Pre-diagnostic phase. AE: angioedema; ICU: intensive care unit

Figure 1 illustrates the journey of the patient who presents repeated angioedema (AE) episodes before HAE-C1INH is diagnosed.

Participants in this study identified several critical points and issues at this stage of the patient journey that may contribute to underdiagnosis or delayed diagnosis of the disease, which are detailed below.

In relation to the number of attacks experienced by patients until diagnosis, the experts noted that they visit hospital EDs or primary care (PC) on repeated occasions until diagnostic confirmation, although this number is difficult to specify since it depends on the patient, the hospital or health care centre he or she attends, and the familiarity of the treating healthcare professional with the disease. Furthermore, in many cases, in the event of a mild-moderate attack, the patient might not go to the hospital ED or PC urgent care [20], remaining at home until symptoms have subsided, so the number of attacks the patient experiences before being diagnosed could be higher than that recorded.

After treatment of an acute attack in the hospital ED or PC, the patient may not be referred to any hospital department. Referral by the ED to allergy/immunology services is not always direct, and the patient is often referred to other departments such as gastroenterology, dermatology, otolaryngology (ear, nose and throat, ENT) or internal medicine. This results in a delay in diagnosis, as well as unnecessary consultations and interventions.

Patient care by allergy/immunology specialists is critical to the diagnosis of HAE. The time to diagnosis is highly variable and, in many cases, very long (an average of 8 years), although this period is longer in the case of hospitals that do not have allergy/immunology departments.

Overall, it was noted that a lack of knowledge about the disease among healthcare professionals seeing these patients results in an unduly long time for referral to allergy/immunology services and, consequently, in a delay in diagnosis.

### Diagnosis of the patient with HAE-C1INH

**Fig. 2:**
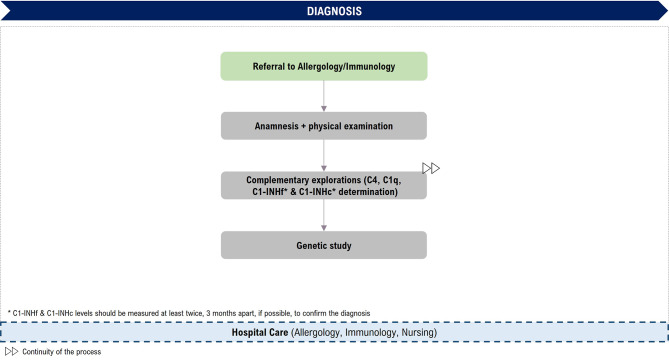
Patient journey: Diagnostic phase. C1q: complement component C1q; C4: complement component 4; C1INHf: C1-inhibitor function; C1INHc: C1-inhibitor concentration

The diagnosis of HAE-C1INH in Spain is generally made by allergy specialists and, in some cases, by immunology specialists. It is based on clinical suspicion in the presence of recurrent and transient episodes of cutaneous AE, abdominal pain and/or upper respiratory tract oedema that do not respond to H1 antihistamines, corticosteroids or adrenaline, along with a family history of AE. However, up to 25% of the *SERPING1* gene are *de novo* mutations and there may be no family history [9]. The diagnosis of HAE-C1INH must be confirmed with laboratory tests. The variability and heterogeneity of the disease make it difficult to confirm the diagnosis, although once the patient is treated by the allergy or immunology department, the time to diagnosis decreases [6]. Figure 2 shows the journey of a patient during the diagnostic phase and details the tests that are generally performed, as defined by the experts.

Experts agreed that referral times are critical to the early diagnosis of HAE-C1INH. In this regard, the delay may be higher in regional hospitals. The tests performed for diagnostic confirmation are similar throughout Spain, although there is greater variability in the case of the genetic study since it is not performed in all hospitals, which could lead to delays in the diagnosis of family members when C1INH results are doubtful.

### Treatment of the patient diagnosed with HAE-C1INH

In recent years, new therapeutic alternatives have emerged for the management of patients with HAE. Pharmacological treatment is currently based on a three-pronged approach: controlling AE attacks (on-demand treatment); preventing the onset of AE attacks with LTP; and preventing the onset of AE in risk situations such as surgical or medical procedures or other stressful situations for the patient with STP [5, 8, 10, 11].

**Fig. 3:**
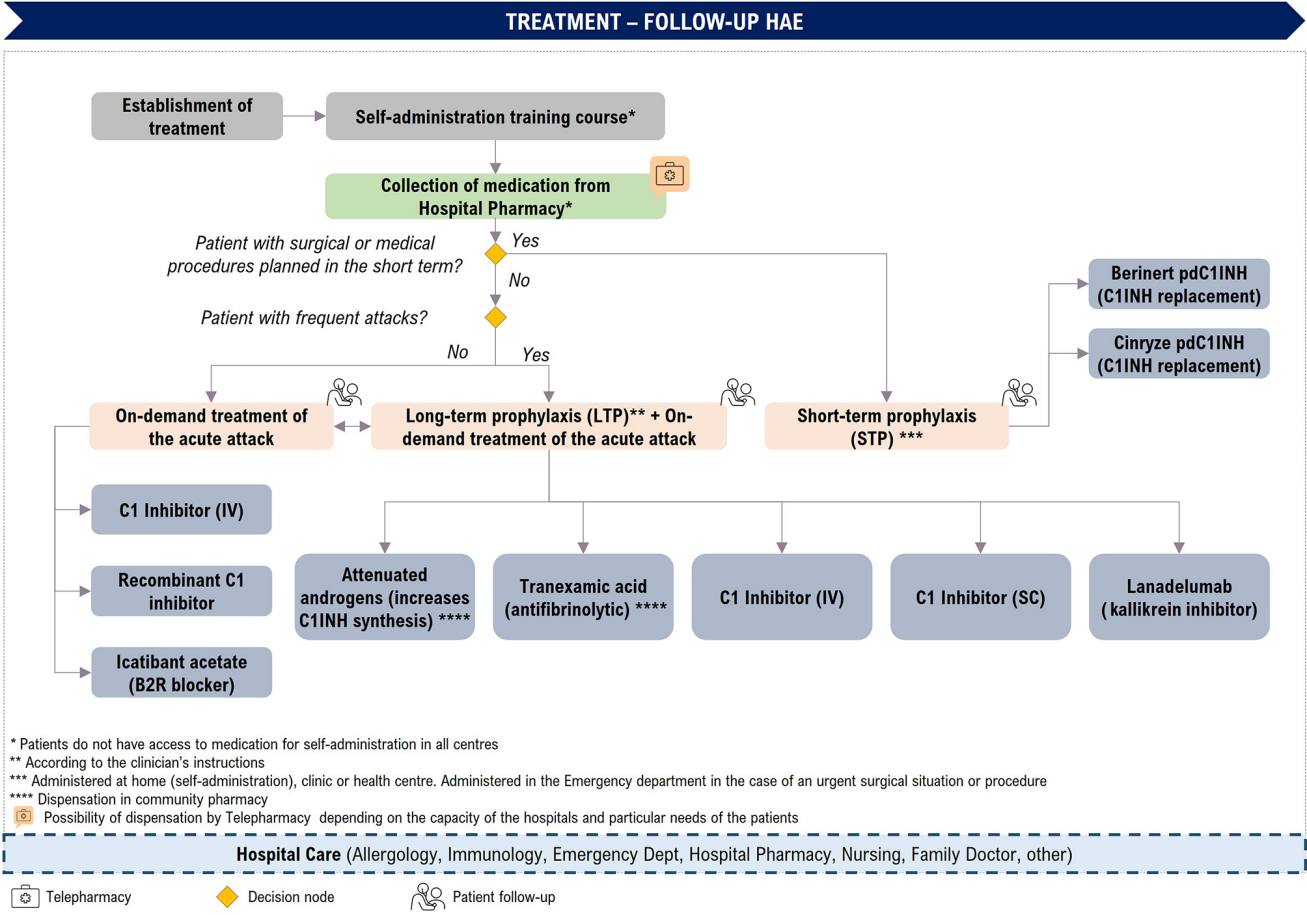
Patient journey: Treatment phase. LTP: long-term prophylaxis; SC: subcutaneous; IV: intravenous; pdC1INH: plasma-derived C1-inhibitor concentrate

The therapeutic strategy is established according to each patient’s condition and will be modified according to the patient’s response and needs at all times. The HAE-C1INH patient journey during the treatment phase has been defined in Fig. 3, detailing each of the mentioned alternatives.

Regarding treatment availability in Spain, HAE-C1INH patients have access to the HAE-C1INH specific drugs through the public hospitals, which fund the drug through their budget.

During this phase of HAE-C1INH treatment, and taking into account the three therapeutic approaches, the experts identified a number of critical points and issues regarding patient management.

Due to the characteristics of the disease, the availability of medication in the patient’s home for self-administration is an increasingly widespread practice that is recommended by guidelines and specialists.

Despite having medication at home, patients sometimes go to a PC centre to have it administered by a healthcare professional, although not all centres provide this service. Self-administration will therefore depends on the patient him- or herself and on the training provided in the hospital.

Lastly, there remain patients who are difficult to control with the current drugs available for HAE-C1INH treatment.

Additionally, the use of telepharmacy programmes has expanded in Spain, particularly after the COVID-19 pandemic, and it is common for patients with HAE-C1INH to be included in such programmes, especially in the case of patients on LTP.

**Fig. 4:**
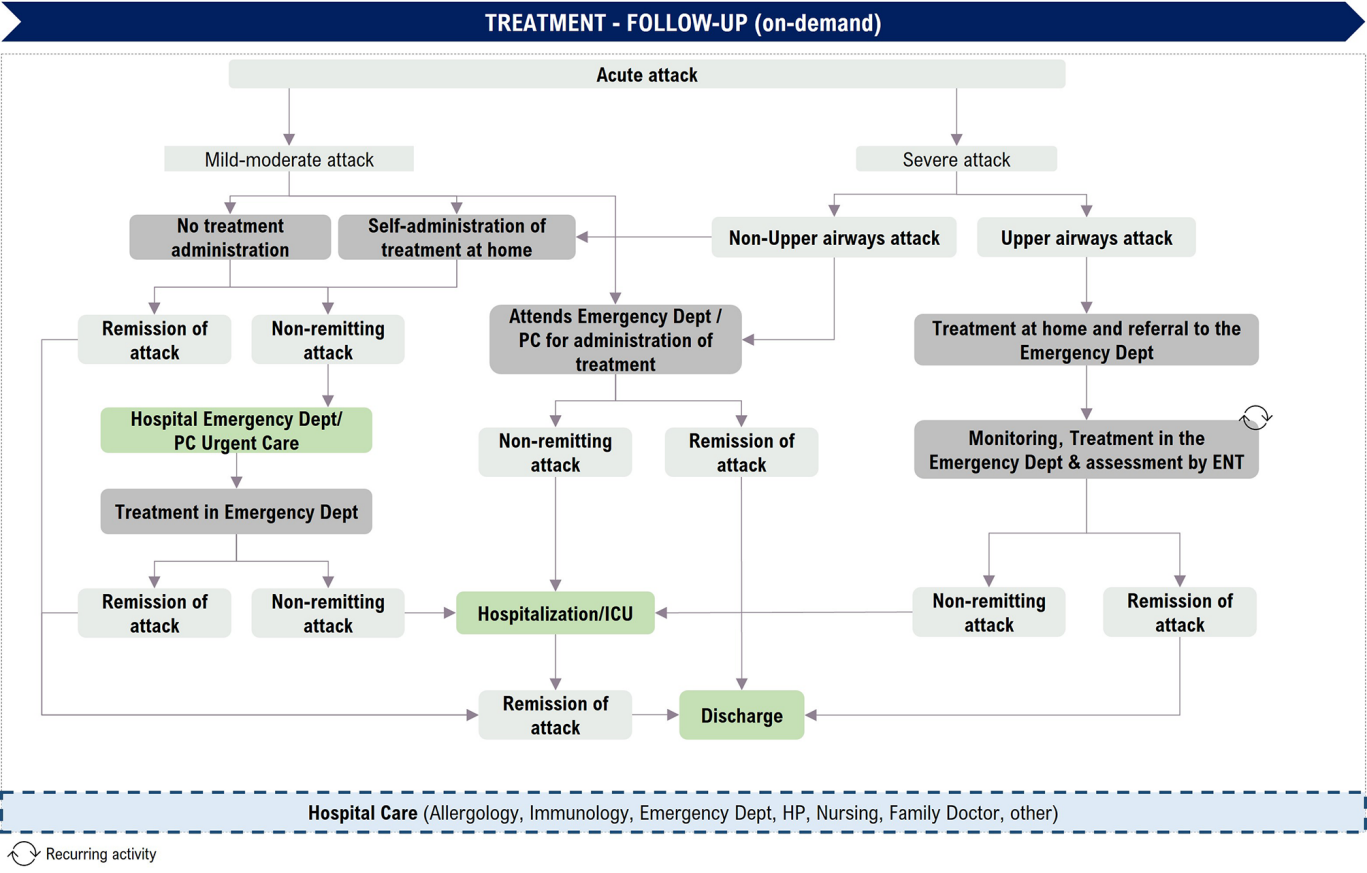
Patient journey: On-demand treatment phase. PC: primary care; URT: upper respiratory tract; ICU: intensive care unit; ENT: Ear, Nose and Throat

The journey of the patient experiencing an acute attack is presented taking into account the differences in terms of the severity of the attack and the location (Fig. 4).

After analysing the HAE-C1INH patient journey the experts identified three areas that generate delays in the healthcare process. Firstly, there are delays on the part of patients in attending primary care. Secondly, there are delays in referring patients from primary care to allergy/immunology departments and then to specialized centres. Finally, the time until obtaining a diagnosis and starting treatment can be optimized.

The experts highlighted the key aspects that should be promoted to optimize the approach to HAE-C1INH.

1. Improve awareness and training on HAE among healthcare professionals, particularly in PC and the ED, as well as in society as a whole.
2. Reduce patient referral times to allergy/immunology.
3. Encourage the conduct of screening studies among the relatives of diagnosed patients.
4. Promote the need for treatment of all acute attacks, both among healthcare professionals and among patients.
5. Ensure equity in the self-administration of treatments for all patients, through systems that facilitate access to medication at home and proper education and training.
6. Ensure equity in access to LTP treatment for all patients who require it.
7. Develop Spanish multidisciplinary consensus that specify the main routes of patient entry and appropriate referral criteria help to reduce variability in HAE-C1INH management in clinical practice.
8. Promote studies that help to increase knowledge of the disease and improve its treatment in accordance with new developments.
9. Promote the collection of data in national registries, generating a greater understanding of the disease and its potential approach.

## Discussion

This is the first study that describes the HAE-C1INH patient journey in Spain. Only a few studies on the patient journey in HAE have been published. The first study was published in 2016 [20] and was performed by a panel of 12 HAE experts and HAE patients from different countries worldwide. The approach was similar to that used in this study and they also identified three stages (1) onset of symptoms and initial evaluation (prediagnostic phase), (2) referral and diagnosis (diagnostic phase), and (3) management of HAE (treatment phase and follow-up). Another study performed in Germany was published in 2020 [21]. They developed a survey that was answered by 81 HAE patients. Finally, a study performed in Mexico has recently been published [22]. In this study, the authors also developed a survey that was answered by 17 HAE patients. In these two last studies, no phases were described in the patient journey.

HAE-C1INH is an underdiagnosed disease with a delayed diagnosis due to the similarity of its symptoms with more common conditions and other factors, such as a lack of awareness on the part of healthcare professionals. Although the time to diagnosis has been reduced in recent years, largely because awareness of the disease has improved, it remains variable and is often longer than desirable, and depends on each patient (number of attacks, location of attack, assigned referral hospital, etc.) [3, 17, 21, 23–25]. Allergy/immunology referral times are considered essential to facilitating early diagnosis of the disease. At present, despite becoming less common, erroneous referrals and unnecessary interventions continue to occur, thus contributing to delayed diagnosis and inadequate patient treatment.

When a series of recurrent AE attacks and the existence of a family history led to suspicion of the disorder, the diagnosis is confirmed by specific laboratory tests. To promote early diagnosis, training on the disease and a push to perform genetic studies have been two of the measures proposed by the experts. The Spanish Society of Allergy and Clinical Immunology (SEAIC) created in 2007 a group of physicians interested in bradykinergic angioedema (GEAB) who developed the first Spanish consensus on bradykinergic angioedema [9, 26]. The Spanish Society of Immunology (SEI by its acronym in Spanish) also formed a group interested in hereditary angioedema (SHINE by its acronym in Spanish) [27]. In addition, EDs are strategic places for the detection and treatment of this disorder; in Spain, the Spanish Society of Emergency Medicine (SEMES) has a specific AE working group with the aim, among others, of training and disseminating knowledge about the disease [28].

Despite guideline and consensus recommendations, real-world clinical practice varies greatly in terms of the management of acute attacks, depending on the patient and the situation. The experts emphasized that the availability of medication in the patient’s home for self-administration is considered essential as any attack can be treated quicker and more effectively, thus improving patient QoL and disease management [5, 8, 10, 11, 26]. In this respect, the experts found that the possibility of HAE patients keeping medication at home varied widely among the different regions and hospitals in Spain, which was confirmed in a survey performed by AEDAF [29].

Another key aspect highlighted by experts was the possibility of self-administration of HAE-C1INH treatments, requiring the patient to keep medication at home. Self-administration allows attacks to be treated quickly, reducing the severity and duration of symptoms, the need for higher treatment doses, and the number of visits to health centres. It also promotes patient autonomy, improving their QoL, and is associated with a reduction in costs, especially in patients who are in areas far from healthcare centres. It is a recommended and increasingly widespread practice in Spain [9, 26, 30] although it is not provided by all centres [29]. Home medication is one of the main demands of patients and healthcare professionals involved in the treatment of HAE-C1INH. Differences in availability to patients vary depending on the healthcare professional, their knowledge of disease management and the healthcare facility (PC, tertiary level hospital ED, regional hospital ED) in which they are located. Self-administration involves training patients and, furthermore, requires their involvement. The publication of an updated national consensus on the management of HAE-C1INH could be of help to increase the number of HAE-C1INH patients having enough medication at home and being able to self-administer the treatment.

Indications on when to treat an acute attack have changed over the course of the different consensuses and guidelines on HAE-C1INH [4, 10, 12, 26, 31–35]. The 2021/2022 International *World Allergy Organization/European Academy of Allergy and Clinical Immunology (WAO/EAACI)* Guideline recommends considering all AE attacks for on-demand treatment, regardless of location, and treating them as early as possible [11]. In the HAE-C1INH patient journey in Spain, many patients do not treat every attack, despite guidelines recommendations. In addition, the International Canadian HAE Guideline [10] states that effective therapies should be used for the acute treatment of AE attacks to reduce their duration and severity. In this HAE-C1INH patient journey in Spain, effective and specific treatments are used for on-demand treatment of angioedema attacks.

For acute attacks with upper respiratory tract involvement, early treatment is critical. For this type of attack, patients are advised to go to the ED after administering medication at home for both monitoring and, if necessary, further treatment [10, 11]. In these cases, medical monitoring is essential to reduce the risk of asphyxia [11, 26]. Nevertheless, despite the recommendations, the experts said that not all patients attend the ED when they present upper respiratory tract involvement.

Moreover, admission to the intensive care unit (ICU) could happen in the case of patients with life-threatening upper respiratory tract involvement, although the experts noted that it has become much less frequent in recent years due to a better understanding of the disease, the identification of patients and new treatments.

Management of the disease has changed in recent years due to the introduction of new therapeutic alternatives that represent an improvement in the approach to patients with HAE. Specifically, the emergence of effective and safe treatments indicated for LTP has led to a paradigm shift in disease management, with the main goals being reaching total control of the disease and normalizing patient life [11]. LTP prescriptions in Spain vary depending on the healthcare professional and the centre in which they practice, and are largely subject to knowledge about the disease, its management and the difficulty of access to treatment due to its high cost. A Spanish consensus on a Treat to Target oriented management of HAE has recently been published [36] and could help to improve the care of HAE-C1INH patients.

The studies in real life and the use of registries could be of great aid in the management of HAE. Different HAE centres in Spain participate in the Icatibant Outcome Survey (IOS) and thus some characteristics of the management of HAE in Spain have been published [3, 30].

The development of new drugs for HAE is a hope for those patients who are difficult to control with the current available drugs [37].

However, as a result of the improved understanding of HAE and new treatments, the management and, therefore, the prognosis are getting better.

The main strength of this study is that it was done by a multidisciplinary team of experts involved in HAE-C1INH diagnosis and management (physicians, pharmacists, nurses), patients, and caregivers. One of the main limitations of our study is the current variability in clinical practice, which depends on the healthcare professional, the specific characteristics of each patient, and the area and hospital to which they belong. This makes it difficult to establish a detailed and homogeneous care pathway for all patients with HAE-C1INH.

## Conclusions

The literature on HAE-C1INH is sparse, largely because it is a rare and poorly understood disease. In this study, in which healthcare professionals of different profiles with experience in the management of patients with HAE-C1INH and the patients themselves participated, the HAE-C1INH patient journey has been detailed at each of the stages of the care pathway, highlighting those key points that could help to improve management of the disease. Moreover, the geographical distribution and the representation of both larger and smaller hospitals in this project afford the study a good perspective of the situation of HAE-C1INH management in Spain.

## Data Availability

All data generated or analysed during this study are included in this published article.
